# Termite Assemblage and Damage on Tree Trunks in Fast-Growing Teak Plantations of Different Age: A Case Study in West Java, Indonesia

**DOI:** 10.3390/insects12040295

**Published:** 2021-03-28

**Authors:** Anugerah Fajar, Setiawan Khoirul Himmi, Astuti Latif, Didi Tarmadi, Titik Kartika, Ikhsan Guswenrivo, Sulaeman Yusuf, Tsuyoshi Yoshimura

**Affiliations:** 1 Research Center for Biomaterials, Indonesian Institute of Sciences (LIPI), Jl. Raya Bogor km. 46 Cibinong, Bogor 16911, Indonesia; didi@biomaterial.lipi.go.id (D.T.); titikkartika@biomaterial.lipi.go.id (T.K.); rivo.guswenrivo@biomaterial.lipi.go.id (I.G.); sulaeman@biomaterial.lipi.go.id (S.Y.); 2 Department of Biology, Faculty of Mathematics and Natural Science, Mulawarman University, Jl. Barong Tongkok No. 4 Gunung Kelua, Samarinda 75242, Indonesia; astutilatif98@gmail.com; 3 Research Institute for Sustainable Humanosphere (RISH), Kyoto University, Gokasho, Uji 611-0011, Japan; tsuyoshi@rish.kyoto-u.ac.jp

**Keywords:** teak plantation, termite infestation, living tree, termite functional group, zeta diversity decline, beta diversity partition, Sørensen index, Jaccard index

## Abstract

**Simple Summary:**

The fact that termites attack healthy living trees is little known, being overshadowed by termite attacks in urban settings. Nonetheless, termites do attack living trees, especially on plantations, and this issue needs greater visibility. Here, we investigate termite attacks on fast-growing teakwood, *Tectona grandis* L.f., and the development of termite communities over time on experimental plantation landscapes located in Cibinong, West Java, Indonesia. Teakwood is a valuable and popular material in the wood industry that has various uses. Our results indicated that termite communities become more complex over time, as demonstrated by the increasing number of species and occupied areas. At the same time, damage observed on tree trunks was limited to bark and was related to the activities of termites that consume fresh biomass, like fungus-growing termites (functional group IIf). In addition, landscapes with overgrown weeds and shrubs were observed to have more damage on tree trunks than did groomed landscapes. Managing understory vegetation might suppress termite attacks on fast-growing teakwood, although we also observed that termite attacks on trunks were inevitable if termites that belong to functional group IIf have managed to occupy large areas.

**Abstract:**

We conducted surveys of termite assemblages and tree trunks damaged by termites in teakwood (*Tectona grandis* L.f.) plantations. The surveys were conducted in five-, six-, and nine-year-old plantations. We used a standardized belt-transect to collect termites and build tree inventories. Data of collected termites at the genus and functional-group levels and termites’ diversity between plantations were compared with their attack rate. The results showed that four genera of soil recycler termites belonging to groups IIf and III were present across the plantations. Distribution analysis suggested that termite communities might develop from a stochastic distribution to a nonrandom co-occurrence distribution over time. Diversity analysis showed an increased nestedness-resultant diversity contribution to the total dissimilarity over time. Observed attacks on tree trunks were superficial and limited to the outer bark, with group IIf as the main contributor. Furthermore, the level of damage done by termites to tree trunks was positively correlated with increases in the group IIf occupancy area and overgrown understory vegetation. Plantation management by maintaining an adequate understory might suppress termite attacks on fast-growing teakwood, although in the case of our study, termite attacks are inevitable when termites from group IIf were already present.

## 1. Introduction

Termites are a dominant group of decomposers in tropical forests [1,2]. Termites are also adaptable and capable of modifying the environments that support them [1]. However, their intrusion into human-managed habitats has led to their status as an economically important urban pest that causes significant damage. Prominent examples include attacks by *Coptotermes gestroi* Wasmann and *C. formosanus* Shiraki [3,4].

Other, less prominent termites are also known to be pests, especially in agricultural/forestry settings, although the damage that they do is hard to estimate [5,6,7]. The appropriate method for assessing termites as potential pests involves determining termite community composition and distribution in hierarchical order, which can reveal termite interaction with the surrounding environment [8,9]. Although the distribution data are rich with information, utilizing them is a complicated endeavor and depends on the nature of the species studied.

It is convenient and cost effective to use incidence-based surveys since abundance-based methods tend to underestimate population size, as many individuals in the juvenile and incipient reproductive castes remain inaccessible to rapid sampling [10,11]. The current development of ecological tools, especially beta diversity partitioning and zeta diversity [12,13,14], allows us to extract and gain new perspectives on species distribution data derived from incidence-based surveys. Beta diversity is commonly used to measure how much one site differs compared to another in terms of species composition [15]. The measurement involves dividing the overall number of available species by the average number of species for each locality in the surveyed area. Zeta diversity, on the other hand, counts the distinct species that are shared among an increasing number of sites [14]. These tools can complement the more common approach used in functional group studies to improve our understanding of the dynamics of termite communities.

Fast-growing teakwood plantations are known to be one of the agricultural landscapes that are vulnerable to termite attack. This understanding grew out of the results of newer studies that found healthy living trees in secondary forests and regenerating woodlands are vulnerable to termite attack [16,17,18]. These findings represent a departure from past opinion that trees attacked by termites were already dying or had become dead stands before the attack [19]. While teakwood is known to have high durability against termite attacks in timber form [20], the durability of teakwood while it is still alive on a plantation is unclear. Past studies have shown that *Neotermes tectonae* Damm. is the main termite that attacks living teakwood stands [21,22]. As fast-growing teakwood was intended to be a substitute for conventional teakwood [20], no information is available on termite profiles in these plantations or on teakwood durability against termite attacks in the plantation or as timber. The only information available thus far is on its mechanical properties. For example, it has been reported that its modulus of rupture (MOR) and wood density at 5 years of age exceed those of conventional teakwood at 10 to 20 years of age [20].

In this study, we investigated fast-growing teakwood plantations aiming to answer two questions: (i) How does the termite assemblage profile develop along with the increasing age of fast-growing teakwood plantations? and (ii) How do existing termite assemblages relate to the threat of teakwood trunk damage by termites on these plantations? The collected information is expected to add to our knowledge of termite communities and the threat of termite infestations over time on fast-growing teakwood plantations.

## 2. Materials and Methods

### 2.1. Sampling Locations

The study was conducted in three different teakwood (*Tectona grandis* L.f.) plantations, which were five, six, and nine years old, respectively. The teak variant used was Platinum Teak, a fast-growing teakwood modified through tissue culture developed by the Research Center for Biotechnology and the Research Center for Biology, Indonesian Institute of Sciences (LIPI) [23,24]. This teak variety is considered promising for furniture products, with five-year-old timber showing characteristics similar to 20–30-year-old conventional teak [20].

The plantations were established in the experimental area in the botanical garden close to the Cibinong Science Center, Bogor, Indonesia, and were approximately 15,000–20,000 m^2^ in size at each location. The land was cleared and fallow before the teakwood was planted. The plantation management involved thinning when the trees reached the ages of 3, 6, and 9 years. At the time of sampling, the plantation details were as follows:

- Five years old (location A): The plantation location was 6°48′84″ S, 106°84′83″ E, at an altitude of approximately 140 m.a.s.l. This was the youngest plantation available. The spacing was 3 m with a moderate canopy cover and a tree height of 6–7 m. The leaf litter was substantial, with almost no herbaceous plants or shrubs present.
- Six years old (location B): The plantation location was 6°50′0″ S, 1065°84′50″ E, at an altitude of approximately 150–145 m.a.s.l. The spacing was 3–4 m with a moderate canopy cover and a tree height of 8–12 m. The herbaceous layer and leaf litter were substantial. Some shrubs were also present.
- Nine years old (location C): The plantation location was 6°49′20″ S, 106°85′60″ E, at an altitude of approximately 135–128 m.a.s.l. The spacing was 4 m with a dense canopy cover and a tree height of 10–15 m. The herbaceous layer and leaf litter were substantial. Some shrubs were also present.

**Figure 1:**
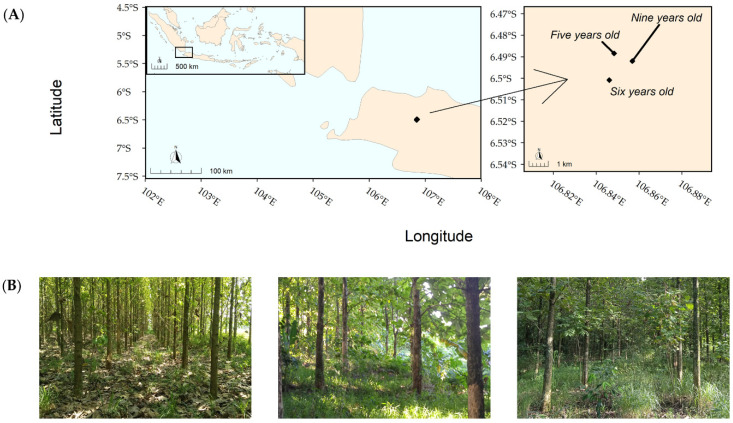
(**A**) Plantation locations in Cibinong, West Java, Indonesia; (**B**) plantation landscapes, from left to right: five-year-old, six-year-old, and nine-year-old plantations.

The area was flat across all plantations. The temperatures ranged from 20 to 35 °C, and the humidity ranged from 60 to 90% all year round. The annual rainfall was around 3456 mm, with >2000 mm of rainfall between November and April [25]. The locations and their conditions are shown below in Figure 1.

### 2.2. Survey of Termites and Damaged Teakwood Trunk

The sampling was conducted in February 2019 using a belt-transect (100 m × 2 m) for each plantation that was placed near the central area to avoid anthropogenic disturbances [26,27,28]. Jones and Eggleton [26] argued that one supervised transect in a surveyed area was sufficient to provide the required level of sampling efficiency for investigating termite assemblage structures [29,30]. The transect was divided into 20 sections; each section was a 5 m × 2 m area. For each designated section, two experienced collectors searched the entire section from 10 cm below the ground up to 2 m above the ground. The collections were completed in less than 30 min for each section. All collected specimens, preferably soldiers, were kept in a vial coded for the corresponding section. A solution containing 70% ethanol was used as a preservative.

Termite damage to teakwood trunks was measured by assessing all existing stands along the belt-transect area. Our survey showed that termite damaged and peeled the trunk bark, leaving a whitish coloring on the trunk. Other than that, there was no damage on the wooden parts, and no noticeable gall growth on the trunks observed. The observed areas of damage were categorized into three levels based on the termite incidence and the accompanying gallery present on the trunk surface (Table 1). The collected data were then tabulated for further processing.

### 2.3. Termite Identification Process

Identification at the species level was performed using morphological characteristics. These were observed using a digital microscope (Leica S8 APO B; Leica Microsystems CMS GmbH, Germany). All observed characteristics were compiled and compared to the guidelines. The primary guideline was Key to the Indomalayan Termites [31], especially the termite soldiers key to families and species level. We also used Termites (*Isoptera*) of Thailand [32], Termites of Sabah [33], and Termites of Peninsular Malaysia [34] for holotype descriptions. All species were deposited at the Research Center for Biomaterials, Indonesian Institute of Sciences (LIPI).

### 2.4. Data Analysis

The identities of the termites were compiled at the species and genus level. The functional group data were based on the termite feeding group as described by Donovan et al. [35], and further refined in Jones and Eggleton [36]. The functional group summary is presented in Table 2.

The incidence of termites at the genera and functional group levels was measured based on the proportion and occupancy of the corresponding termites. Both measurements were based on relative abundance [26]. The proportion was calculated as the share of the entire termite population made up by each genus and functional group. The proportion was calculated by tallying each encounter with the same genus or functional group and then dividing it by the total number of encounters with all genera or functional groups observed during the survey. The occupancy was measured by counting the termites spread over the plot by adding the incidences of each genus or functional group across all 20 sections and then dividing by 20.

The damage to the tree trunks was assessed based on the criteria listed in Table 1, and the number of damaged trunks at each level were then tallied. The number of damaged trunks at each level was then divided by the total number of trees along the transect belt and transformed into a percentage. The percentage of Level 3 damage to the trunk and the termites’ occupied area at the functional group level were also depicted using a linear model, and then the model fitness was determined using the least-squares method.

The assessment of termite distribution was made using zeta diversity and beta diversity. For the zeta diversity, we computed the number of distinct species that were shared among each successive addition of the transect section, known as the zeta diversity number, while the number of those of the transect sites is known as the zeta diversity order. We did the calculation for up to five pooled sections from the total of 20 sections in a transect belt using the zeta diversity decline function from the zetadiv R package [38].

The number of distinct species of the zeta diversity was then plotted in two regression models. For the exponential model, the number of distinct species was transformed into a log scale and plotted along the *y*-axis with the untransformed number of sections along the *x*-axis. For the power-law model, the number of sections was also transformed into a log scale before being plotted along the *x*-axis. The curve fitness to these two regressions was then evaluated using the Akaike information criterion (AIC), with lower AIC values indicating better fitness to one of the regression models [14,39]. According to Hui et al. [14], better fitness to the exponential model indicated a species distribution that happened stochastically, while better fitness to the power-law model indicated a distribution driven by niche differentiation processes [14,40].

To further verify the zeta diversity, the species accumulation curve for each plantation was also constructed using EstimateS (Version 9.1, R. K. Colwell, http://purl.oclc.org/estimates) (accessed on 1 April 2019) with 100 randomizations with replacements for curve smoothing. We present the curves in the supporting materials (Figure S1).

For the beta diversity, we partitioned the beta diversity of the Sørensen and Jaccard indices into turnover (species replacement) and a nestedness-resultant dissimilarity component [12,41]. The partitioning was done at the multisite level (uppercase subscript notations) and at the pairwise level (lowercase subscript notations). We also measured the ratio of nestedness/(nestedness + turnover) for the Sørensen and Jaccard indices at the multisite (β_RAT_) and pairwise (β_rat_) levels to assess the diversity differences between plantations [41]. The beta diversity partitioning was calculated using the betapart R package with 100 randomizations to generate the beta score [42].

Most of the calculations were made in R (Version 3.6.2, R Core Team, https://www.R-project.org/) (accessed on 25 February 2020), and the figures were generated using the ggplot2 R package [43] and the rnaturalearthdata R package (Version 1.0, A. South, https://cran.r-project.org/package=rnaturalearth) (accessed on 3 March 2020).

## 3. Results

### 3.1. Termite Identity, Functional Group, and Termite Damage on the Trunks

Samplings from the three plantations yielded six termite species from the five- and six-year-old plantations, and nine species from the nine-year-old plantation. All recovered specimens belonged to four genera, three of which were from group IIf and one from group III. The four genera that were recovered were found throughout the plantations studied. In total, 13 species were collected, of which three species were found at all plantations. A summary of the species identities is presented in Table 3.

**Figure 2:**
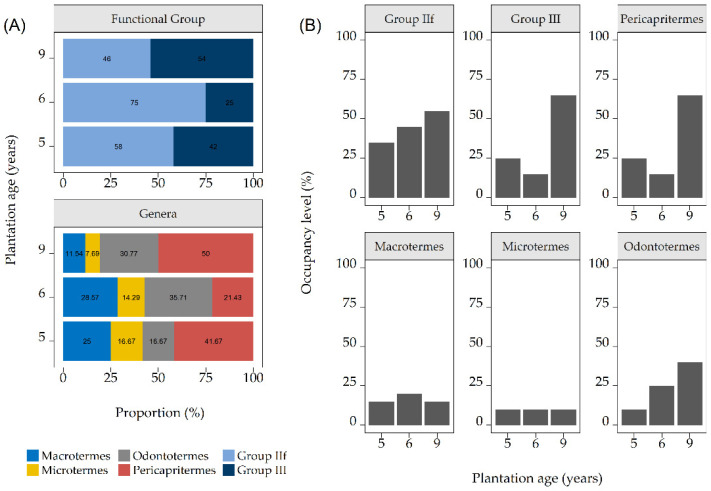
Termite assemblage based on (**A**) proportion and (**B**) occupancy in three teakwood plantations. The denominators used to calculate the proportions were the total numbers of genera and functional groups observed during the study; for occupancy, it was the total number of plot sections per plantation (20 sections).

The data on the proportions and occupancy of the termite genera and functional group distribution across the teakwood plantations are depicted in Figure 2.

The proportion assessment results shown in Figure 2A were as follows. (i) The *Odontotermes* and *Pericapritermes* proportions in the nine-year-old plot were 2–3 times larger than in the five- and six-year-old plots, while those of *Macrotermes* and *Microtermes* were smaller in the nine-year-old plot compared to the five- and six-year-old plots. (ii) In group IIf, only *Odontotermes* increased in proportion over time, suggesting that *Odontotermes* had a higher probability of being recovered compared to other genera from IIf. (iii) In the six- and nine-year-old plots, the combined totals of the proportions of group IIf genera (78.57% and 50%, respectively) were larger than those of the IIf functional groups (75% and 46%, respectively), while in the five-year-old plot this difference was much smaller, with the combined proportions being 58.34% for the genera and 58% for the functional groups. These findings suggest the presence of a higher number of plots being shared by group IIf species in that area.

Several important observations regarding occupancy (Figure 2B) were made, as follows. (i) The occupancy levels of *Macrotermes* and *Microtermes* were relatively constant across the plantation age groups. (ii) Group IIf had the highest occupancy level at the nine-year-old plantation, although the proportion data indicated otherwise. This result revealed that group IIf expansion was outrun by group III at the nine-year-old plantation. (iii) The number of plots occupied by termites increased over time for both functional groups, indicating positive growth in the foraging areas of termite communities, especially for group III.

**Figure 3:**
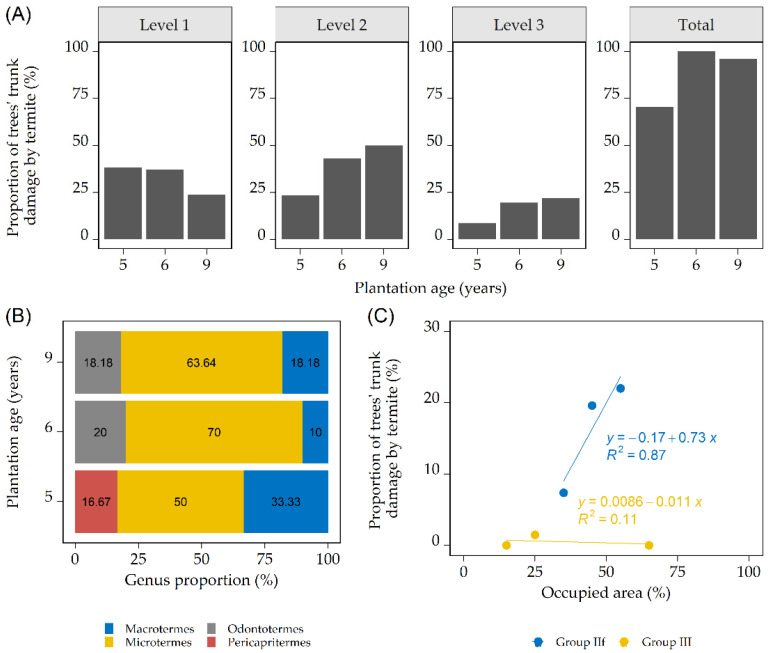
Termite attacks on teakwood trunks. (**A**) Attacks at all three levels of damage and the combined damage from Level 1 to 3; (**B**) the termites collected from teakwood trunks with Level 3 damage; and (**C**) relationship between functional group occupied area and active termite incidence on teakwood trunks with Level 3 damage. See Table 1 for a description of the damage levels caused by termites (Levels 1, 2, and 3).

In terms of termite attacks on teakwood trunks, the study collected 68 trees, 51 trees, and 50 trees for the five-, six-, and nine-year-old plantations, respectively (Figure 3).

The data in Figure 3A show that there was a decrease in Level 1 damage and increases in Levels 2 and 3 along the plantation age. The data also show that the total number of trunks damaged by termites was higher in the six- and nine-year-old plantations than in the five-year-old plantation, which is interesting since the five- and six-year-old plantations are closer in age to each other than they are to the nine-year-old plantation. The most common termites that caused damage on trunks were from group IIf, with *Microtermes* as the biggest contributor (Figure 3B). The relationship between the occupied area and termite attacks (Figure 3C) indicated that group IIf termites attacked trees and were positively correlated with the increase in the occupancy area; it should be noted that this is based on a small amount of data.

Visual inspection indicated that the damage did not go any further than the inner bark of the trees, as indicated by un-barked sections of trunks with some evident indentations and with group IIf termites found on the trees. On the other hand, group III termites were found between tree roots in small chambers and under leaf litter. No clear signs of damage caused by group III were found except for one occurrence on a trunk’s outer bark right above the ground in the five-year-old plantation.

### 3.2. Termite Distribution and Diversity

**Figure 4:**
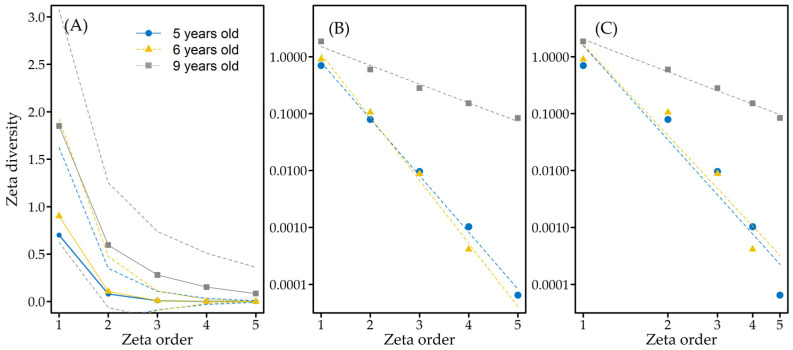
Zeta diversity results with (**A**) number of distinct species (zeta diversity) shared by one to five sections, here known as the zeta order; and the distribution model fitness of (**B**) exponential fitting for the independent/stochastic distribution; and (**C**) power-law fitting for dependent/niche-driven distribution for each plantation. The dashed line in 4A indicated the 95% confidence interval of the zeta diversity.

The termite distribution as indicated by zeta diversity analysis (Figure 4A) showed no significant difference at 95% confidence in the number of species shared from one section to five sections of the transect belt.

The results showed that, on average, the five- and six-year-old plantations only contained species that were shared among up to three sections, fewer than in nine-year-old plantations (Figure 4A). The model fitness showed that five- and six-year-old plantations were better fitted to the exponential model (AIC exp: −5.299 and −1.11) than the power-law model (AIC pl: 10.578 and 8.725). In comparison, the nine-year-old plantation had mixed results, with fitness similar to the power-law and exponential models (AIC pl −11.47 and AIC exp: −6.83) (Figure 4B,C). These results suggested that the distribution of the termites developed from a stochastic distribution in the younger plantations to a distribution that was driven by species interaction in older plantations.

**Figure 5:**
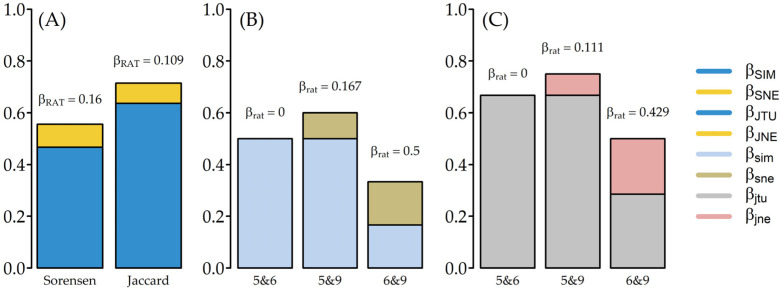
Beta diversity partitioning at (**A**) the multisite level using the Sørensen index with turnover (β_SIM_) and nestedness (β_SNE_) and the Jaccard index with turnover (β_JTU_) and nestedness (β_JNE_); (**B**) Sørensen pairwise level with turnover (β_sim_) and nestedness (β_sne_); (**C**) Jaccard pairwise level with turnover (β_jtu_) and nestedness (β_jne_). The values of β_RAT_ and β_rat_ were calculated as the ratio of nestedness to the total nestedness plus turnover. The pairwise comparison label format, e.g., 5&6 = five-year-old plantation compared to six-year-old plantation.

The beta diversity from the Sørensen and Jaccard indices (Figure 5A) at the multisite level showed that the nestedness component made little contribution to diversity dissimilarity across plantations, as indicated by the small β_RAT_ for both.

The in-depth beta diversity partition of the pairwise comparison revealed several interesting observations, as follows. (i) The contribution of nestedness-resultant diversity to the total diversity dissimilarity increased along the plantation age, as indicated by the increase of the β_rat_ score, starting from zero nestedness-resultant diversity between the five- and six-year-old plantations to diversity dissimilarity of almost 50%in the six- and nine-year-old plantations (Figure 5B,C). (ii) In terms of nestedness-resultant diversity (Figure 5B,C), the five- and six-year-old plantations were subsets of the nine-year-old plantation, but the five-year-old plantation was not a subset of the six-year-old plantation, although three species were shared between the two plantations. (iii) The overall diversity dissimilarity indicated that termite assemblage in the five-year-old plantation was the most dissimilar compared to those in the other plantations. Following these three observations, visual inspection across the plantations showed that the five-year-old plantation had a minimal understory, while the six- and nine-year-old plantations had overgrown understories (see Materials and Methods).

## 4. Discussion

In the present study, the functional groups recovered from the surveyed teakwood plantations were limited to groups IIf and III. A similar distribution was also observed in earlier surveys of agricultural/forestry areas in Southeast Asia. In those surveys, group I was usually recovered in disturbed environments, while group II was found in recently or selectively logged plantations [30,44]. These past studies also reported that group III and sometimes group IV were usually absent from the inventory where landscape burning had been used to clear the area, and they may only recover much later via colonization from an unburned adjacent area [30,45]. Our finding of group III could be attributed to the absence of landscape burning and to the land having been fallow before the teak trees were planted.

The absence of group I can be attributed to the maintenance of the plantations, such as through thinning. The thinning process ensures that dead trunks, which are common dietary materials for group I [46,47], were cleared from the plantations. The wood removed through thinning was immediately cleared from the site and sold to a nearby wood processor. With regard to the dry-wood termite *N. tectonae* (group I), we did not recover them in our study, but it has been reported to specifically attack living teakwood in a past study [48]. The local climate may have an effect on the absence of *N. tectonae* in the present study. The past studies were located in areas with more defined seasonal weather, and were also drier, than the locality investigated in the present study [21,22,25,49]. Additionally, we did not recover any *Nasutitermes* species (group II), while some past studies on young plantations (<10 years old) reported the presence of this genus [30,44,50]. This difference can be attributed to the plantations’ past land-use history. Our surveyed plantations were formerly agricultural fields, while those in the previous studies were primary forest before being converted into plantations [30,44]. Additionally, we may have missed some termite species due to limited transects we conducted.

In the present study, we investigated the dynamic between termite functional groups in teakwood plantations along with the increase in the plantation age and the damage they caused to tree trunks. The results indicated that the trunk damage was caused by termites from specific functional groups. Previous studies of termite distribution showed that competition for the same dietary resources or the development of specialization to a specific dietary resource is one of the main drivers in shaping the distribution of termites and the impact they have on the environment [46,51].

Reviewing past studies on the foraging habits of termites [46,52,53] has revealed that the observed functional group composition emerged due to the adaptation of the termites to the available dietary material. Termite functional groups are sorted based on their adaptation to the level of dietary substrate humification [46]. Group IIf termites use fresh litter biomass, a high-carbon, high-energy source that is available around trees, for their gardening activities [54,55], while most genera in group III use only litter biomass with a high degree of decomposition, which has low carbon and energy contents compared to fresh litter biomass [35,46,56]. These adaptations cause group III to have scattered colonies that are interconnected to one another and keep expanding over time to compensate for their low-energy diet, which contrasts with the localized spread of group IIf termites around trees, as indicated by the presence of galleries and runways on teakwood trunks.

The division between groups IIf and III on the basis of substrate humification is reflected in our surveys of damaged trunks, in which group IIf emerged as the main cause of damage due to the availability of outer bark as a fresh litter source (Figure 3B). The emergence of group IIf is reflected in the positive correlation between the areas occupied by group IIf and the incidence of group IIf termites on tree trunks (Figure 3C). We also noted that group IIf attacks were only found in the stem bark, and they left the wood components unharmed except for some serration and dents on the sapwood surface. The consumption of bark was probably motivated by its higher nitrogen content compared to other wood components, as demonstrated in the gallery development of *Gnathamitermes tubiformans* Buckley [57]. On the other hand, the dietary substrate for group III was mainly found in spaces between tree roots and decomposed leaf litter, which is why the group III feeding activities did not cause damage to tree trunks on a large scale like the activities of group IIf.

Other than the composition of termite assemblages, the overall distribution and diversity reflected in the present study suggested, albeit loosely due to the low species richness, that the species distribution of termites becomes more complex and abundant as the age of the plantation increases. This result was similar to the finding of a study of termite biodiversity in eucalyptus plantations [58]. However, according to the zeta diversity, the development of the distribution was not correlated with the amount of trunk damage since the total damage already peaked at 100% in the six-year-old plantation (Figure 3A) when the distribution was still stochastic (Figure 5C). As a reference, the six- and nine-year-old plantations shared similar amounts of trunk damage (Figure 3A) while they had different models of termite distribution.

Interestingly, beta diversity partitioning showed that almost half of the dissimilarity in diversity between the six- and nine-year-old plantations was contributed through nestedness-resultant diversity, which was something that five- and six-year-old plantations did not share. Further field observation showed that five-and six-year-old plantations shared the landscape conditions as the nested feature (see the Sampling Locations section and Figure 1), which indicated habitat nestedness [59,60,61].

The functional groups found showed that the trunk damage was not equally caused by all termites present in the plantations. Only group IIf was observed as the main viable threat of trunk damage (Figure 3C). Thus, a small change in diversity dissimilarity, e.g., the absence of group IIf in one area, can result in a completely different profile of tree trunk damage in that area compared to others in which group IIf was present. Furthermore, the presence of an intact gallery on a tree trunk without any termites recovered (Damage Level 2, see Table 1) may indicate that there might be a recurring infestation on the tree trunk or a hidden infestation underground since these galleries indicated recent foraging activity. However, a hidden infestation that might be present in the root system may have gone unrecorded, since we only dug ~10 cm below the ground during the transect.

The three recovered genera from group IIf (Table 3) were known for their foraging galleries made of soil sheeting on plant surfaces. These galleries were used to cover the biomass of the plants being consumed. However, *Microtermes* in particular was also known for attacking trees from their roots and tunneling to the stem with the leftover cavity filled in by soil [5,62,63]. While our study showed that *Microtermes* was found on the trunk surface under the soil sheeting, no tunneling or indentation on the wooden components was observed.

The genus *Pericapritermes* (group III) showed a high occupancy area across the plantations in the current study (Figure 2), and yet we only recorded one occurrence of *Pericapritermes* on the damaged trunk areas. The small amount of trunk damage caused by *Pericapritermes* can be attributed to the bark substrate, which was different from the dietary preference of group III [46,51]. Interestingly, past studies have demonstrated that *Pericapritermes* attacked the tap roots of young trees in *Eucalyptus* sp. and *Shorea* spp. [64,65]. Earlier studies also reported that *Pericapritermes* galleries together with their foragers were found next to tree fibrous roots in rhizospheres [56]. Therefore, *Pericapritermes* may pose a potential threat to teakwood trees in the future and should be mitigated if necessary.

The landscape conditions were likely to have a major effect on the amount of trunk damage, as suggested by the trunk damage counts (Figure 3) and the beta diversity (Figure 5). We noted that the five-year-old plantation was narrower in terms of the general spacing between trees compared to the other plantations. An earlier study on teakwood planting indicated that having closer spacing between trees creates a tighter canopy that causes the understory to die due to a lack of sunlight [66], which leads to the almost complete absence of a layer of herbaceous plants and shrubs in the five-year-old plantation. The absence of herbaceous plants and shrubs will expose termites’ covered runways on teakwood trunks to external disturbances, such as being washed away by rainfall [67] or direct human intervention [62]. Because the soil sheeting is easily destroyed, termite damage on the teakwood trunk is reduced.

Termite damage to tree bark cannot be overlooked. Tree bark is vital since it protects the phloem layer, which is the vascular tissue in charge of the transporting and distribution of organic nutrients. Scratches or damage on the bark can damage the tender phloem layer, which can cause tree death or open a pathway for tree diseases [68]. To mitigate trunk bark damage, we believe that conducting periodic clearing of the understory, herbaceous plants, and shrubs is a viable way to mitigate trunk infestation by termites. This method has been recommended by field guidelines for teakwood planting in Indonesia [66]. The method exposes termite galleries and runways to outside disturbances, which discourages termites from expanding their foraging areas. It also deprives existing termites of resources, even though it disrupts the soil nutrient cycling facilitated by termites [1,2]. In addition, excessive clearing can cause a loss of soil through surface runoff, according to an earlier study [67].

Overall, the trunk damage done by termites at our study sites was superficial, indicating that the hazard caused by the termites present on fast-growing teakwood in the current case study is minimal. On the other hand, questions remain as to what kind of further damage can be caused by groups IIf and III if more changes were to occur between the age groups in our study. Further studies are necessary to obtain a better understanding of termite assemblages and the trunk damage they cause in teakwood plantations larger than those in our current study, as upscaling the size would increase the complexity of the termites’ community, distribution, and diversity.

## 5. Conclusions

We investigated termite attacks on living teakwood trunks and the existing termite assemblages on three plantations with a progressively increasing plantation age. We discovered four genera from two functional groups (IIf and III), with group III more distributed in older plantations than group IIf. We also found that termite communities develop a more complex distribution over time, with species starting to fit into an ecological niche as time passes. Meanwhile, the beta diversity partition indicated that the diversity was mainly derived from species replacement, but nestedness-resultant diversity also increased over time, with landscape conditions as the nested feature. The damage done by termites to tree trunks was almost exclusively done by group IIf. Furthermore, our results indicated that an increase in the termite occupancy areas was positively correlated with an increase in termite attacks on trees. We also observed that differences in landscape conditions might inhibit the damage done by termites to teakwood.

## Figures and Tables

**Table 1:** Damage level of teakwood trunks caused by termites based on the bark condition.

Damage Level	Teakwood Bark Condition
Level 1	The trace of termite damage on the bark is observed, but neither gallery nor termites are present.
Level 2	The bark has been disturbed and peeled, with a gallery observed but no termites present.
Level 3	The bark has been disturbed and peeled, with both a gallery and termites present.

**Table 2:** Termite functional groups based on feeding habits [27,35,36,37].

Feeding Group	Feeding Habit
I	Deadwood and grass feeders. The only group with flagellate protists in their guts
II	Feed on miscellaneous types of grass, dead wood, leaf litter, and micro-epiphytes
IIf	Feed on grass, dead wood, and leaf litter, with the help of fungal symbionts grown inside the nest (fungus-growing termites)
III	Feed on organically rich upper soil layers (humus feeders)
IV	Feed on organically impoverished soil or mineral soil (true soil feeders)

Note. The entire list of genera and the corresponding functional groups are provided in the Appendix of Donovan [35] and in Table 17.2 of Jones and Eggleton [36].

**Table 3:** Species identity and richness from three platinum teakwood plantations. (A = 5 years, B = 6 years, C = 9 years after establishment).

Family/Sub-Family	Genus	Species	Functional Group	Incidence Presence		
				A	B	C
Macro-termitinae	*Macrotermes*	*Macrotermes gilvus*	IIf	✓	✓	✓
	*Microtermes*	*Microtermes insperatus*	IIf	✓	✓	✓
	*Odontotermes*	*Odontotermes grandiceps*	IIf	✓	✓	✓
		*Odontotermes javanicus*			✓	✓
		*Odontotermes karnyi*				✓
		*Odontotermes bilitoni*				✓
		*Odontotermes longignathus*				✓
		*Odontotermes sp1* ^a^			✓	
Miro-capritermitinae	*Pericapritermes*	*Pericapritermes semarangi*	III	✓		
		*Pericapritermes mohri*			✓	✓
		*Pericapritermes latignathus*				✓
		*Pericapritermes A* ^b^		✓		
		*Pericapritermes B* ^b^		✓		
Species Richness				6	6	9
α diversity = 23/3; γ diversity = 13; β diversity = 39/23						

^a^ The species is unknown; the species is close to *O. grandiceps* size-wise, but had a more rectangular head compared to *O. grandiceps.* ^b^ The species matched the description given by Tho [34], although Tho himself did not name the species and only used a designated letter as a marker.

## Data Availability

The data presented in this study are available on request from the corresponding author.

## Supplementary Materials

The following are available online at https://www.mdpi.com/article/10.3390/insects12040295/s1, Figure S1: Species accumulation curve (SAC) from three teak plantations of different ages.
