# Supplementary material for: Termite Assemblage and Damage on Tree Trunks in Fast-Growing Teak Plantations of Different Age: A Case Study in West Java, Indonesia
Source: Insects. 2021 Mar 28;12(4):295. doi: 10.3390/insects12040295 (PMC8067069; doi:10.3390/insects12040295)
Supplement: Supplementary file 1 [file insects-12-00295-s001.pdf]

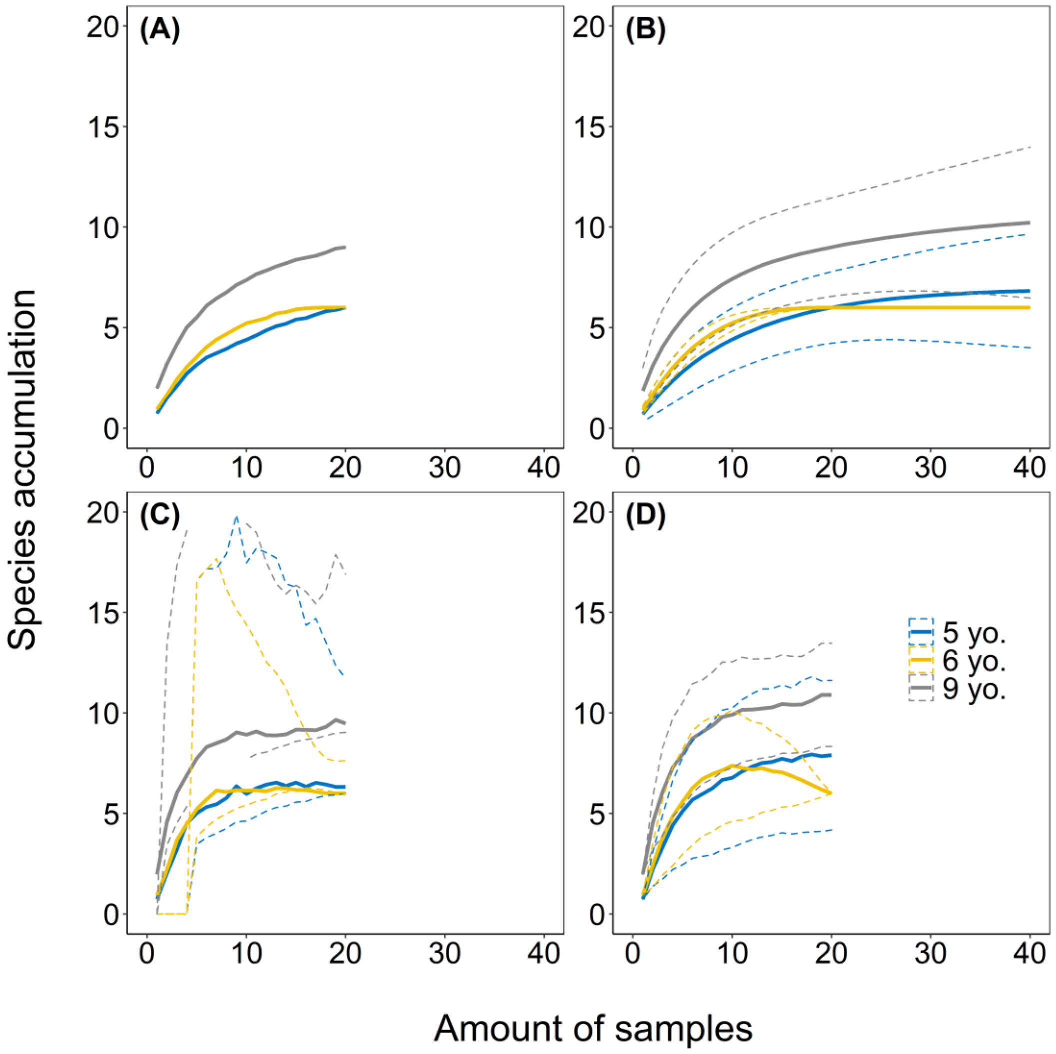

Figure S1. Species accumulation curve (SAC) from three teakwood plantations of different age. (**A**) original mean of species accumulation curve for 20 samples and (**B**) Bernoulli product extrapolation (hypothetical sample from number 21 to 40). The estimator curve for 20 samples of (**C**) Chao 2 and (**D**) Jackknife 1. Each estimator was shown to include confidence interval at 95% indicated by dashed line.
